# Evaluation of Electrospun PCL-PLGA for Sustained Delivery of Kartogenin

**DOI:** 10.3390/molecules27123739

**Published:** 2022-06-10

**Authors:** Steven Elder, John Graham Roberson, James Warren, Robert Lawson, Daniel Young, Sean Stokes, Matthew K. Ross

**Affiliations:** 1 Department of Agricultural & Biological Engineering, James Worth Bagley College of Engineering, Mississippi State University, Starkville, MS 39762, USA; jgr222@msstate.edu (J.G.R.); jdw1149@msstate.edu (J.W.); 2 Department of Biochemistry, Molecular Biology, Entomology and Plant Pathology, College of Agriculture & Life Sciences, Mississippi State University, Starkville, MS 39762, USA; rl1054@msstate.edu; 3 Department of Comparative Biomedical Sciences, College of Veterinary Medicine, Mississippi State University, Starkville, MS 39762, USA; dry38@msstate.edu (D.Y.); mross@cvm.msstate.edu (M.K.R.); 4 Department of Chemistry, College of Arts and Sciences, Mississippi State University, Starkville, MS 39762, USA; sstokes@chemistry.msstate.edu

**Keywords:** kartogenin, electrospinning, polycaprolactone, poly(lactic-co-glycolic acid)

## Abstract

In this study, kartogenin was incorporated into an electrospun blend of polycaprolactone and poly(lactic-co-glycolic acid) (1:1) to determine the feasibility of this system for sustained drug delivery. Kartogenin is a small-molecule drug that could enhance the outcome of microfracture, a cartilage restoration procedure, by selectively stimulating chondrogenic differentiation of endogenous bone marrow mesenchymal stem cells. Experimental results showed that kartogenin did not affect the electrospinnability of the polymer blend, and it had negligible effects on fiber morphology and scaffold mechanical properties. The loading efficiency of kartogenin into electrospun membranes was nearly 100%, and no evidence of chemical reaction between kartogenin and the polymers was detected by Fourier transform infrared spectroscopy. Analysis of the released drug using high-performance liquid chromatography–photodiode array detection indicated an abundance of kartogenin and only a small amount of its major hydrolysis product. Kartogenin displayed a typical biphasic release profile, with approximately 30% being released within 24 h followed by a much slower, constant rate of release up to 28 days. Although additional development is needed to tune the release kinetics and address issues common to electrospun scaffolds (e.g., high fiber density), the results of this study demonstrated that a scaffold electrospun from biodegradable synthetic polymers is a suitable kartogenin delivery vehicle.

## 1. Introduction

An isolated cartilage lesion is a common arthroscopic finding [1], and it can have a profound detrimental impact on a patient’s quality of life due to the associated pain and functional impairment [2]. Symptomatic patients frequently require surgical treatment because the intrinsic repair capacity of articular cartilage is extremely limited. Total knee arthroplasty (TKA) is generally successful, with 85–96% implant survival during the second decade of follow-up [3]. However, a study of TKA in patients 55 years old or younger found that only 10% returned to regular recreational or competitive athletic activity [4]. Thus, there is an urgent need for efficacious cartilage regeneration procedures to delay or prevent TKA in younger patients.

Bone marrow stimulation, such as microfracture (MFx), is by far the most commonly performed procedure to treat focal lesions [5]. Compared to autologous chondrocyte implantation (ACI) and matrix-assisted chondrocyte implantation (MACI), MFx is appealing because it is a simple, single-stage procedure. ACI and MACI both require a minor surgery to harvest cartilage and a second surgery to implant expanded chondrocytes. On the other hand, MFx entails debridement and perforation of the subchondral bone plate in a single surgery. The perforations provide pluripotent bone marrow mesenchymal stem cells (BMSCs) in the marrow cavity access to the defect, where they become entrapped in a fibrin clot, differentiate, and give rise to fibrocartilage. Whereas type II collagen represents 90–95% of the collagen in hyaline cartilage, fibrocartilage consists of predominantly type I collagen and has less than half the compressive stiffness [6]. MFx-induced repair tissue is also deficient in proteoglycan [7], well known for its contribution to compressive resistance. Due to its limitations, MFx is considered to be a *good* treatment option only for lesions <2 cm^2^ [8]. It is contraindicated when the defect is ≥4 cm^2^ [9]. Furthermore, a systematic review concluded that treatment failure after MFx could be expected beyond 5 years post-op [10]. Thus, fibrocartilaginous repair has limited durability.

Our long-term goal is to develop a bioactive scaffold which can be applied as an augmentation to MFx to reliably stimulate the regeneration of hyaline cartilage with greater durability than fibrocartilage. The formation of a high proportion of hyaline cartilage may also increase the size of the lesion that can be effectively treated using MFx. The reasons that MFx results in fibrocartilage include insufficiency of BMSCs, inadequate scaffolding for the BMSCs, and a lack of chondrogenic differentiation factors [11]. The purpose of the current study is to describe a membrane designed to overcome these limitations when secured into the base of a microfractured defect using fibrin glue. It is fabricated by electrospinning a blend of polycaprolactone, poly(lactic-co-glycolic acid), and kartogenin (PCL-PLGA-KGN).

Electrospinning yields nanofibers with an interconnected porous structure and large surface area to volume ratio. In vivo, BMSCs would be expected to migrate and proliferate throughout the scaffold, leading to uniform defect filling. In addition, drugs may be loaded into electrospun nanofibers with high efficiency and released in a sustained manner. The drug of interest is KGN, a small molecule that selectively stimulates the chondrogenic differentiation of BMSCs without inducing hypertrophy [12]. It has been shown to promote the regeneration of cartilage with abundant type II collagen and glycosaminoglycan (GAG) [13], as well as protect normal cartilage in mechanically destabilized joints [12,13,14,15,16]. KGN upregulates chondrogenic gene expression through activation of the Runt-related transcription factor 1 (RUNX1) pathway [17]. An advantage of KGN over growth factors such as transforming growth factor-β3 is that KGN is very stable and may be stored and transported at room temperature [13]. Therefore, the use of KGN allows for the fabrication of a bioactive scaffold with potential for long-term storage at room temperature. The potential for KGN to increase hyaline cartilage formation after MFx has already been demonstrated. Twelve weeks after surgery on rabbits, cartilage defects treated by MFx plus weekly intra-articular injection of KGN displayed better defect filling with more hyaline-like tissue compared to the solvent control [18]. We hypothesize that sustained delivery of KGN will elicit a more reliable and robust response. Application of a KGN-releasing membrane as an augmentation to MFx is intended to obviate the need for culture-expanded or intra-operatively isolated BMSCs. We envision a nanofibrous scaffold promoting *endogenous* BMSC distribution to every part of the defect, where they undergo KGN-induced chondrogenic differentiation.

The overall objective of this study was to determine the feasibility of using a PCL-PLGA electrospun membrane to sustain the release of KGN. Specific objectives included determining the effect of KGN on electrospinning and vice versa, as well as KGN loading efficiency and release kinetics.

## 2. Results

### 2.1. Electrospinning, Morphology, and Water Contact Angle

**Figure 1:**
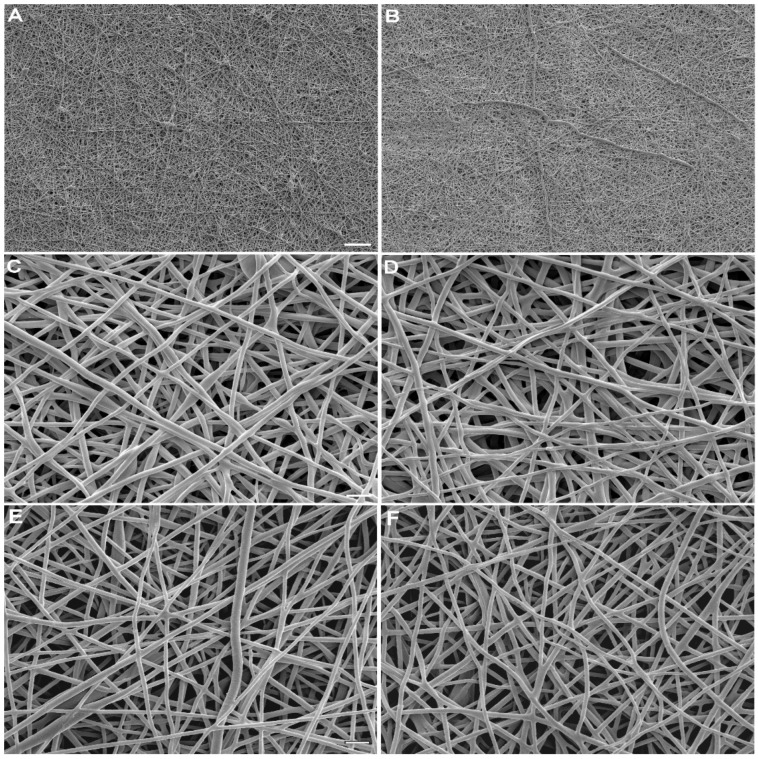
Representative scanning electron micrographs of electrospun 12% PCL-PLGA (1:1): (**A**,**C**,**E**) control scaffolds contained no KGN and (**B**,**D**,**F**) experimental scaffolds contained 16.7 mg of KGN per gram of polymer blend. (**A**–**D**) Meshes are shown as fabricated and (**E,F**) after 28 days of soaking in PBS. Scale bar: (**A**,**B**) 10 μm; (**C**–**F**) 1 μm.

With respect to electrospinning, PCL-PLGA behaved the same whether or not KGN had been added. The formation of a Taylor cone and stable nanofiber jet was readily achieved regardless of the addition of KGN. Resulting membranes were similar in macro and microscopic appearance. They all contained smooth fibers, some of which were fused, and some of which were beaded (Figure 1). KGN did not affect mean fiber diameter, which was 225 ± 24 nm for KGN and 227 ± 5 nm for the control (*p* = 0.88). Fiber diameter slightly increased during soaking in PBS for 28 days to 232 ± 20 and 238 ± 8 for KGN and the control, respectively. Again, the difference was not statistically significant (*p* = 0.54). Likewise, wettability was not sensitive to the presence of KGN. Water contact angles for KGN and the control were 120.7 ± 2.9° and 122.5 ± 1.9°, respectively (*p* = 0.13).

### 2.2. Tensile Properties

**Figure 2:**
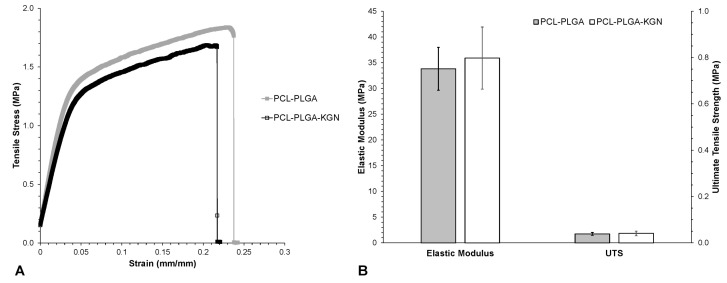
Mechanical properties of electrospun PCL-PLGA with and without KGN: (**A**) representative stress–strain curves and (**B**) material properties. Differences between control and KGN were not statistically significant. Error bars = ± one standard deviation.

Control and KGN-loaded membranes displayed a similar resistance to tensile loading. They were linearly elastic up to about 2% strain, after which the stiffness decreased (Figure 2A), presumably as fibers underwent significant realignment to the direction of the applied load [19]. Necking of the samples was observed at one end or the other, and failure consistently occurred at the same end near the edge of the clamp. Control and KGN membranes were flexible and tough, stretching more than 20% before rupture (Figure 2A). KGN scaffolds were approximately as stiff and strong as controls; slight differences were not statistically significant (*p* > 0.45 for modulus and UTS) (Figure 2B). Uncertainty in the thickness undoubtedly contributed to the observed variability in mechanical properties.

### 2.3. Loading Efficiency and FTIR

**Figure 3:**
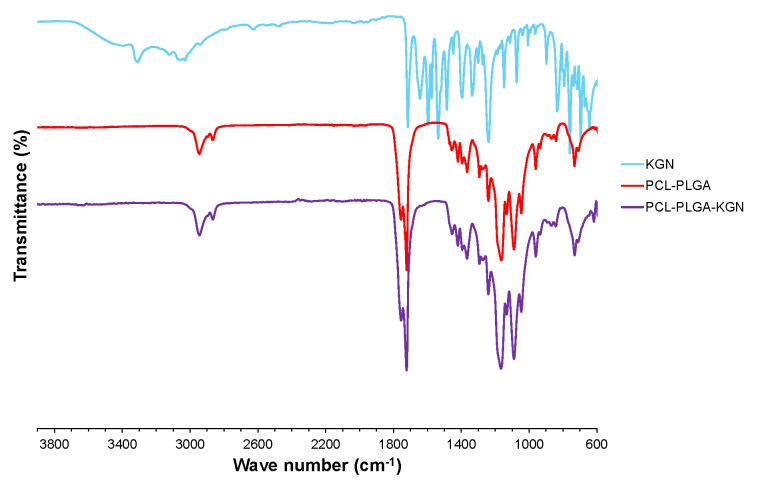
FTIR spectra of pure KGN (powdered form), PCL-PLGA blend (electrospun mesh), and PCL-PLGA-KGN composite (electrospun mesh).

Because KGN loading is a simple matter of adding it to the electrospinning solvent, it is theoretically possible to load KGN into PCL-PLGA scaffolds with a very high efficiency. Measurement confirmed this theory, indicating a KGN loading efficiency of 97 ± 16%. FTIR analysis was performed to determine whether KGN chemically reacted with PCL-PLGA (Figure 3). The FTIR spectrum of the PCL-PLGA that was electrospun in the presence of KGN showed no characteristic absorptions, indicating that the KGN had modified the polymer [14,16,20]. The carbonyl stretching frequencies for the esters found in PCL-PLGA (1722 and 1735 cm^−1^) and PCL-PLGA-KGN (1722 and 1735 cm^−1^) confirmed no deviation of the absorption when compared to the carbonyl of the carboxylic acid in pure KGN (1715 cm^−1^). Additionally, the absence of the OH stretch (broadening between 2800 and 3700 cm^−1^), the NH stretch (3305 cm^−1^), the =C-H stretch (3050 cm^−1^), the amide carbonyl stretch (1642 cm^−1^), and the aromatic ring stretches (1536–1594 cm^−1^) in the PCL-PLGA-KGN spectrum helps illustrate that KGN formed no new chemical linkages to PCL-PLGA.

### 2.4. KGN Release Kinetics and Degradation

**Figure 4:**
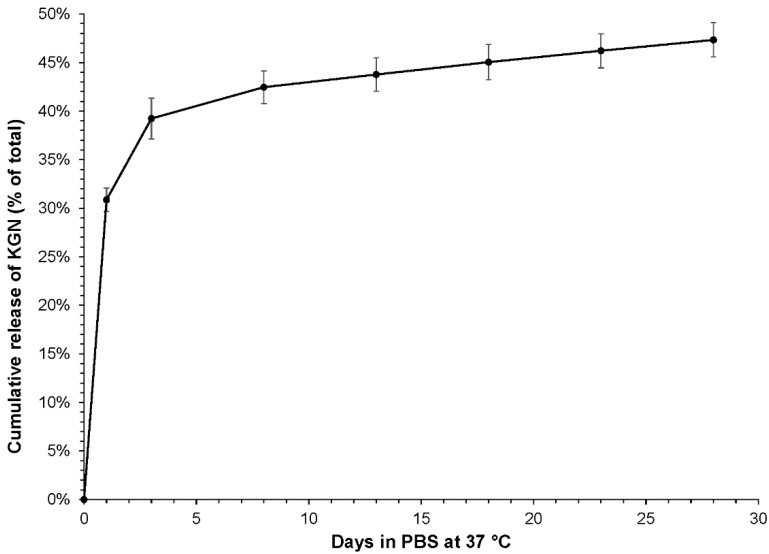
Kinetics of KGN release from PCL-PLGA (1:1) electrospun mesh into PBS. Conditions were 37 °C and orbital shaking at approximately 100 rpm.

The kinetics of KGN release are displayed in Figure 4. A burst release occurred within 24 h, after which the rate of release slowed dramatically. From Day 8 onwards, the rate of release was steady at 0.24% per day. At the end of the 28-day release period, the samples contained, on average, 50% of the total amount of KGN loaded.

**Figure 5:**
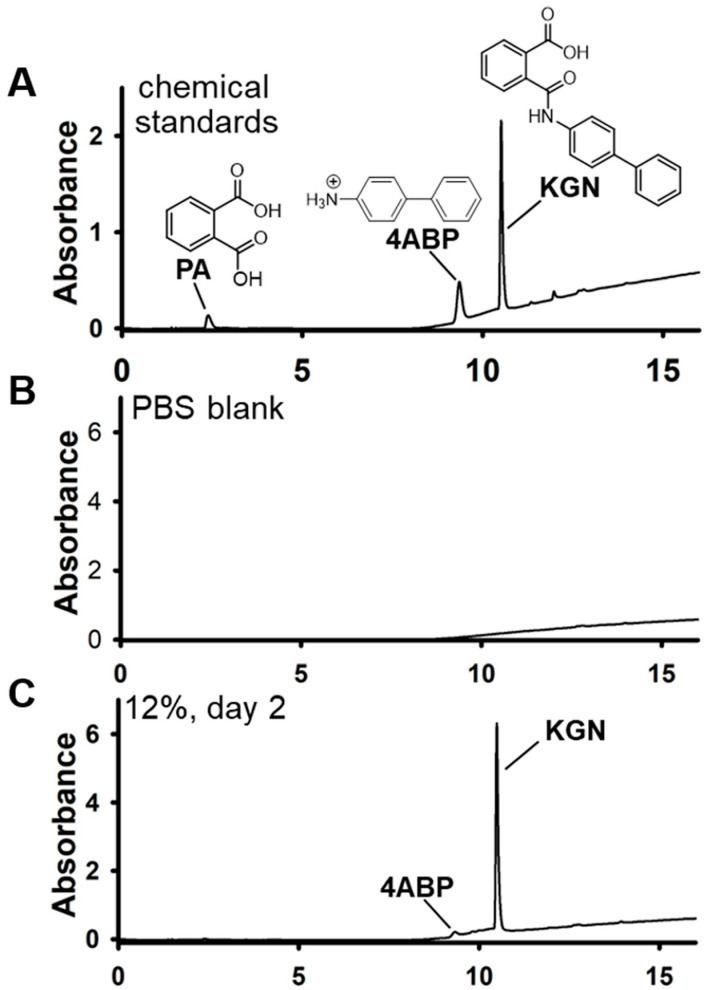
Reversed-phase HPLC-PDA chromatograms of soaked scaffolds. PDA parameters: 200–350 nm. (**A**) Elution profile of the chemical standards (Sigma): PA, phthalic acid; 4ABP, 4-aminobiphenyl; KGN, kartogenin. (**B**) PBS blank extract (negative control). (**C**) The 12% scaffold, day 2 extract. For the day 2 extracts, KGN accounted for >90% of the peaks detected by the PDA detector. Details of the extraction procedure are described in the Materials and Methods.

HPLC–PDA was used to determine the effects of electrospinning and prolonged scaffold soaking in PBS on KGN chemical integrity (Figure 5). At the Day 3 time point, KGN accounted for approximately 94% of the UV-absorbable material detected, while 4-ABP essentially made up the rest (6%).

## 3. Discussion

MFx opens channels in the subchondral bone which provide access to undifferentiated mesenchymal cells from the bone marrow. It has great appeal for being a simple means of bringing endogenous repair cells to the site of a cartilage injury in a one-step procedure. In fact, MFx is unique among the surgical options for the operative management of focal chondral lesions because it leverages endogenous mesenchymal stem cells in a single-stage procedure without the need for isolation or concentration of cells from bone marrow, adipose tissue, or peripheral blood. Because it is simple and minimally invasive, MFx is performed much more frequently than restorative procedures such as autograft, allograft, and ACI. A study of over 500,000 patients found that arthroscopy with debridement/shaving of articular cartilage and MFx accounted for 95.6% of all chondral procedures performed in 2014, with 45.0% of them being MFx [21]. All other chondral procedures combined accounted for just 4.4%. The purpose of this study was to begin development of a kartogenin-loaded, electrospun scaffold for use as an augmentation to MFx that can stimulate hyaline cartilage regeneration. It is intended to obviate the need for culture-expanded or intra-operatively isolated BMSCs. In theory, the scaffold would promote *endogenous* BMSC distribution to every part of the defect, and their exposure to KGN would induce chondrogenic differentiation.

The major drawback to MFx is that the repair is mechanically inferior fibrocartilage, which is distinguished from native cartilage by increased cellularity, a preponderance of type I collagen (rather than type II), and a lack of zonal organization through the depth. It is considered to be a first-line procedure because the repair has limited durability. For example, repair survival in a prospective cohort of 119 patients treated by the MFx technique had fallen below 60% within 3 years [22]. Enhancement of marrow stimulation such that it reliably leads to hyaline cartilage, rather than fibrocartilage, would not only make it a favorable one-step procedure for treating sizable cartilage lesions, but could also greatly increase the repair’s longevity.

Scaffolds can help to guide tissue formation in three dimensions, and acellular scaffolds alone have the potential for modest improvement to the quality of MFx-mediated cartilage repair. Augmentation of MFx with atelocollagen gel resulted in a significantly higher type II:type I collagen ratio one year after surgery, as well as a better MOCART score, compared to microfracture alone [23]. Similarly, defect filling and functional outcomes of hyaluronic acid-based cell-free scaffold-augmented MFx were somewhat better 1 and 2 years after surgery versus microfracture alone [24]. However, the sustained delivery of KGN can have a profound impact. Twelve weeks after surgery on rabbits, cartilage defects treated by MFx plus weekly intra-articular injection of KGN displayed almost 100% defect filling with hyaline cartilage-like tissue (e.g., strong type II collagen immunoreactivity and almost no type I collagen staining) [18]. By contrast, defects in joints injected with the KGN solvent alone were approximately 50% filled with tissue that stained intensely for type I collagen and barely at all for type II. In a rabbit model, Li et al. demonstrated that KGN-releasing thermogel applied to a microfractured defect supported the regeneration of cartilage that was biochemically and histologically similar to native articular cartilage [13].

The seminal work of Johnson and co-workers identified KGN as a drug-like small molecule which promotes the chondrogenic differentiation of BMSCs [12]. It has been shown to promote the regeneration of cartilage with abundant type II collagen and glycosaminoglycan (GAG) [13], as well as protect normal cartilage in mechanically destabilized joints. [12,13,14,15,16] KGN upregulates chondrogenic gene expression through activation of the Runt-related transcription factor 1 (RUNX1) pathway [17]. KGN’s chondroinductive properties are comparable to those of transforming growth factor-β3 (TGF-β3) with respect to the upregulation of SOX9, ACAN, and COL2A1, but KGN does not upregulate the hypertrophic marker COL10A1 as TGF-β3 does [25]. Stability is another advantage of KGN; it is very stable and may be stored and transported at room temperature [13]. The use of KGN allows for the fabrication of an affordable, bioactive scaffold with the potential for long-term storage at room temperature.

The scaffold of interest in this study was an electrospun membrane. Electrospinning yields nanofibers with an interconnected porous structure and large surface area to volume ratio. In vivo, BMSCs would be expected to migrate and proliferate throughout the scaffold, leading to uniform defect filling. A synthetic polymer was selected so that the degradation and release of KGN would occur in a reliable and predictable fashion. PCL is frequently employed for cartilage tissue engineering, mainly because it retains its structure in physiological solutions and its slow degradation rate is compatible with the slow process of cartilage regeneration [26]. However, PCL may take longer than 2 years to degrade in vivo, and there was concern over its suitability to deliver a chondroinductive substance by hydrolysis-mediated release [27]. Hence the decision to blend it with 50:50 PLGA, which also undergoes bulk degradation but is almost completely degraded within 2 months [28,29]. Advantages of blending PLGA with PCL are better wettability, mechanical properties, and cell proliferation [30,31].

The results of this study demonstrate that KGN, when it constitutes approximately 2% of the final mass, has no effect on the ease with which PCL-PLGA can be electrospun, nor does it significantly affect the electrospun membrane’s fiber morphology, wettability, or tensile properties. The resulting membranes were strong enough to be handled without concern over tearing, and their flexibility would have facilitated conforming to the contours of a human joint surface. The elastic modulus and strength of the 50/50 PCL/PLGA scaffold were somewhat lower than the values of 70 MPa and 3 MPa, respectively, which have been reported for a 40/60 blend [19]. However, those scaffolds were produced from a 15% *w*/*v* solution, were composed of much larger fibers, and were cut into a dog-bone shape for testing. In another study, electrospinning of a 70/30 PCL/PLGA solution (12% *w*/*v* PCL, 10% *w*/*v* PLGA) produced scaffolds with fused fibers ranging in diameter from 500 to 5000 nm (50% of which were around 2000 nm) and tensile strength of approximately 3.5 MPa [19]. Differences in starting materials may also have been a contributing factor with respect to both studies; for example, PLGA (85:15) was used in the latter, as opposed to PLGA (50:50) in the current study.

As expected, KGN could be loaded into the electrospun scaffold with nearly 100% efficiency. Furthermore, the process is extremely simple owing to the solubility of KGN in DMF, a component of the solvent used for PCL and PLGA. The solvent is evaporated during the electrospinning process, leaving behind PCL, PLGA, and KGN blended evenly together. Previously published FTIR analysis has shown that PCL and PLGA do not chemically react and that the resulting fibers are simply blended composites [19]. The FTIR analysis in this study confirmed the previous result and produced no evidence of a reaction between KGN and PCL-PGLA. Thus, KGN release likely occurs mainly through the mechanisms of diffusion and hydrolytic polymer degradation [32]. In a study describing the patterns of hydrophobic dexamethasone release from PCL and PLGA (50:50) meshes, rapid release of 98% of the drug was observed to occur within 24 h from PCL meshes [33]. By contrast, only 39% had released from PLGA meshes within 3 days, and the early, sharp release was followed by a sustained release through day 28. These findings would suggest that the rapid release of hydrophobic KGN in this study occurred mainly from PCL with a minor contribution from PLGA, and that sustained release was supported by the hydrolysis of PLGA. Indeed, PCL is known to be highly permeable to small drug molecules [34].

Although the 1:1 blend of PCL and PLGA resulted in sustained release of KGN, the kinetics were less than ideal due to the excessive loss of drug through the initial burst release. Future directions will include the evaluation of several approaches to improve release kinetics, such as lowering the PCL content. However, pure PLGA meshes are not suitable due to substantial macroscopic shrinkage which occurs within a day of soaking in PBS [33]. Based on the previous study of dexamethasone, a hydrophobic small molecule such as KGN, increasing the PLGA lactide-to-glycolide ratio to 85:15, or substituting pure polylactide for PLGA are also promising strategies for achieving sustained release with minimal burst from electrospun meshes [33]. The current study does not address factors other than drug delivery which could limit the clinical application of an electrospun mesh serving as a cell scaffold, and additional development will address hydrophobicity and small pore size, which could limit cell adhesion and migration.

## 4. Materials and Methods

PCL (Capa^®^ 6500), 50,000 MW was from Ingevity Corporation (North Charleston, SC, USA), and PLGA was LACTEL ester-terminated, 50:50 poly(DL-lactide-co-glycolide), 127,500 MW, from Evonik Corporation (Birmingham, AL, USA). Kartogenin was from Shaanxi Dideu Medichem Co., Ltd. (Xi’an, Shaanxi, China). Solvents were from Sigma-Aldrich (St. Louis, MO, USA). A custom electrospinning apparatus was comprised mainly of a GenieTouch Syringe Pump from Kent Scientific (Torrington, CT, USA) and an ES30P-5W 0–30 kV variable power supply from Gamma High Voltage Research, Inc. (Ormond Beach, FL, USA).

### 4.1. Electrospinning

Dimethyl formamide (DMF) was added dropwise to 1,1,1,3,3,3-hexafluoro-2-propanol (HFP) under electromagnetic stirring at a ratio of 9:1 HFP:DMF. If KGN was included, then it was first dissolved in DMF before mixing with HFP. PCL was added to HFP/DMF at 12% *wt*/*v*, and PLGA was likewise added to HFP/DMF at 12% *wt*/*v* in a separate container. Once the polymers had dissolved, they were thoroughly blended 1:1 for at least 24 h by gentle rocking. The final concentration was 20.8 mg of KGN per gram of polymer blend. Electrospinning was carried out for approximately 5 h at a 0.75 mL/h flow rate and a 20 cm needle-to-collector distance. The collector was a 15 cm^2^ flat copper plate covered with a thin sheet of aluminum. Voltage was adjusted to achieve the formation of a Taylor cone and stable nanofibrous jet, which occurred at approximately 15 kV. Two membranes containing KGN were fabricated, as well as two controls without KGN.

### 4.2. Membrane Characterization

Two samples from remote locations on each membrane were plasma sputter-coated with platinum and imaged on a JEOL 6500F Field Emission Scanning Electron Microscope (Peabody, MA, USA). Three images per sample were captured, and the average fiber diameter in each image was determined using SIMPoly, a MATLAB-based image analysis tool [35]. SEM imaging was performed in the same manner for samples which had soaked in PBS for 28 days. A custom apparatus was used to measure the water contact angle as an indicator of membrane hydrophilicity/hydrophobicity. Using a CCD camera with a macro lens, the profile of a 5 µL sessile drop of distilled water was captured immediately after it was deposited onto the membrane. Water contact angles were determined using the Contact Angle plug-in to ImageJ. Membrane mechanical properties were determined by tensile testing. Strips of membrane 60 L × 4 W mm were cut using a scalpel blade. Precise width was found through computerized measurement of seven manually drawn lines using ImageJ. Thickness at three evenly-spaced locations was determined under a force of 0.01 N applied to a 2 mm flat indenter using a Biomomentum Mach-1 Micromechanical System (Laval, Québec, Canada), the same system used for tensile testing. Based on differences in the thicknesses measured at each location, uncertainty in the true thickness is estimated at 0.02 mm (membranes were typically around 0.15 mm thick). The ends of each strip were wrapped with laboratory tape and gripped such that the exposed length was approximately 35 mm. Exact gauge length was taken as the grip-to-grip separation under a preload of 0.1 N. Strips were then pulled to failure at a constant rate of 0.05 mm∙s^−1^. Elastic modulus, ultimate tensile strength, and elongation at rupture were determined from the resulting stress–strain curve (*n* = 7 per group).

### 4.3. Kartogenin Chemical Integrity, Loading Efficiency, and Release

Possible chemical interaction between KGN and the synthetic polymers was investigated by means of Fourier-transform infrared spectroscopy (FTIR). Spectra for KGN (as manufactured), PCL-PLGA electrospun mesh, and PCL-PLGA-KGN electrospun mesh were measured over a wavelength range of 4000–400 cm^−1^ using a Thermo Scientific Nicolet iS5 FTIR spectrometer with an Attenuated Total Reflectance (ATR) sampling accessory (Waltham, MA, USA). Components were analyzed individually as manufactured (pellet or powder) and also combined as electrospun membranes. The loading efficiency of KGN into electrospun scaffolds was calculated as in Equation (1):

(1) $$KGN\;Loading\;Efficiency=\frac{KGN_{calc}-KGN_{meas}}{KGN_{calc}}$$

where *KGN_calc_* is the KGN concentration found multiplying the KGN known mass fraction by the sample weight and *KGN_meas_* is the concentration determined from spectrophotometry. To create the standard curve of absorbance versus KGN concentration in HFP:DMF, a typical PCL-PLGA electrospinning solution (total polymer concentration 12% *wt*/*v*) was diluted 1:15 in the HPF:DMF electrospinning solvent. Additional standards were generated by a series of two-fold dilutions in HFP:DMF (9:1). Using a Thermo Scientific NanoDrop 2000C, peak absorbance was determined to occur at 268 nm. A268 was plotted against concentration, and the curve was linear up to 0.13 mM. Pieces of KGN-loaded membrane were weighed and re-dissolved in HFP:DMF (9:1), and the standard curve was used to determine the amount of KGN present in each sample after diluting in HFP:DMF as necessary to bring the concentration within the linear region. All readings were taken after blanking with the diluted electrospinning solution containing no KGN.

The kinetics of KGN release in vitro were determined by soaking in phosphate-buffered saline (PBS) at 37 °C under gentle agitation. Triplicate samples of membrane each weighing approximately 13 mg were incubated in 1 mL of PBS. At time intervals of 1, 3, 8, 13, 18, 23, and 28 days, the PBS was collected and replaced with fresh PBS. The amount of KGN released into the PBS was determined by measuring the absorbance at 280 nm and comparing it to a standard curve generated by dissolving KGN directly into PBS. At the end of the 28-day release period, the samples were air dried and dissolved in HFP:DMF to quantify the amount of KGN remaining.

The effect of electrospinning and soaking in PBS on KGN chemical integrity was analyzed using high-performance liquid chromatography–photodiode array (HPLC–PDA). A portion of the PBS soaking solution from Day 3, which had been replaced at the end of Day 1, was collected and stored frozen at −80 °C. Thawed solution was extracted using C18 SepPak columns (60 mg). After adding the sample (1 mL) and washing the column with 95:5 *v*/*v* water:methanol + 0.1% acetic acid, the analytes were eluted in 2.5 mL of methanol. Eluate was evaporated under nitrogen and the residues reconstituted in 100 µL of methanol for HPLC-PDA analysis (200–350 nm). KGN and 4-aminobiphenyl standards were similarly analyzed. 4-aminobiphenyl is the major KGN hydrolysis product [15].

### 4.4. Statistics

Quantitative data were analyzed using *t*-tests (assuming unequal variances) in Microsoft Excel, with the significance threshold set at *p* = 0.05.

## 5. Conclusions

The addition of KGN to PCL and PLGA does not interfere with the electrospinning process, nor does it affect the morphology or mechanical properties of the electrospun mesh. Loading efficiency approaches 100%, and KGN does not appear to chemically react with the synthetic polymers. KGN released from electrospun meshes soaked in PBS underwent minimal degradation, and the release kinetics followed a biphasic pattern of a burst followed by a slow, sustained release. Overall, the results of this study suggest that electrospun PCL-PLGA is a suitable vehicle for the sustained delivery of KGN. Although the release kinetics of the tested formulation are not ideal, the system presents several opportunities for improvement.

## Data Availability

Data is contained within the article.

## Sample Availability

Samples of the compounds are not available from the authors.
